# Systematic reviews and meta-analyses during the SARS-CoV-2 pandemic

**DOI:** 10.31744/einstein_journal/2022CE0041

**Published:** 2022-06-21

**Authors:** Lucas Sousa Maia Ferros, Gustavo Gonçalves Yogolare, Sergio Carlos Nahas, Francisco Tustumi

**Affiliations:** 1 Faculdade de Medicina Universidade de São Paulo São Paulo SP Brazil Faculdade de Medicina, Universidade de São Paulo, São Paulo, SP, Brazil.; 2 Hospital Israelita Albert Einstein São Paulo SP Brazil Hospital Israelita Albert Einstein, São Paulo, SP, Brazil.

Dear Editor,

The article “Meta-analyses on COVID-19: scoping review and quality analysis”,^(1)^ published in this journal, shows its relevance when planning meta-analysis and carrying out pertinent discussion about the role of this type of methodology in the current pandemic.

An essential instrument of evidence-based medicine, meta-analysis represents the top of the scale of level of evidence and grade of recommendation.^(2)^However, the emergency pandemic situation caused by the severe acute respiratory syndrome coronavirus 2 (SARS-CoV-2), identified in January 2020, brought several implications related to how we conduct, read and analyze systematic reviews and meta-analyses, since there were no original studies on the novel disease.

The lack of original studies while searching for the best evidence for decision-making, together with the high transmission rate and severity of the disease, influenced the use of non-traditional databases, such as preprint bases. Available updated materials are crucial elements in a disease that progresses daily. Preprint databases aimed to accelerate the reporting of research data, making it easier to gather information, but not relying on peer review. The absence of peer review makes publication of preprints susceptible of erroneous analysis,^(3)^ such as the manuscript on reduction of hospital morbidity by use of hydroxychloroquine plus azithromycin, which was withdrawn by the authors after the controversy over the study design. Conventional databases, such as MEDLINE and Embase almost exclusively have articles published after peer review; however, they may take months to be published. As *e.g.,* the media preprints database had most of the original works related to coronavirus disease 2019 (COVID-19) included in 2021.^(4)^

In addition, systematic reviews on SARS-CoV-2 quickly become outdated, favoring greater use of peculiar forms of review, including “rapid reviews”, “living systematic reviews”, and “scoping reviews”.

The rapid review is a synthesis of knowledge that aims to provide information with greater speed as compared to systematic reviews. In this process, the components are omitted or simplified to provide faster information for decision-making. There is a usual selection of works already known in the environment and the reviewer chooses the steps that will be limited, and later provide explanation with the effect of the methodological choice. However, this methodology allows for much bias when compared to systematic reviews, for simplifying this process.^(5)^

In general, a rapid review takes approximately four months or less and is considered useful in emergency situations, such as epidemics and disaster relief. As an example, the study by Wu et al.^(6)^ provided a comprehensive overview of the safety profile of COVID-19 vaccines using rapid review. A systematic review, however, usually takes from 12 to 24 months to be carried out and is preferred in situations where evidence will be used to inform decisions, or in developing guidelines that will be implemented in large scale.

Another type of review that gained notoriety during the pandemic is the living systematic review. This is a systematic review that is frequently updated, including new relevant evidence as it emerges and keeping review findings constantly updated. It uses standard systematic review methods and maintains an explicit and *a priori* commitment to a predetermined frequency of research and review updates. In this sense, live systematic reviews are most appropriate when the field of research covered by the systematic review is moving quickly and new evidence is emerging, as observed with COVID-19. Siemieniuk et al.^(7)^ performed a living systematic review on drug treatments for COVID-19, comparing the effects on the disease. Nonetheless, this type of study demands a continuous and active workflow, with coordinated effort over extended periods by the review team, searching for new articles.^(8,9)^

Pires et al.^(1)^ published a scoping review. This type of review is a research synthesis that aims to map the existing literature on a particular topic or research area to identify key concepts and knowledge gaps. It has a comprehensive character, and during the current pandemic, it has been useful to provide the attending physicians with the fundamental concepts on management of COVID-19. On the other hand, scoping review can often present generic ideas and requires physicians to investigate more about the topic, searching for other sources of evidence for a more informed approach.^(10,11)^

It is worth mentioning these changes in the profile of the main review articles derived from the COVID-19 pandemic require a more critical view of readers and physicians. Critical evaluation is an evidence-based medicine tool for judging methodological deficiencies. The judgment of a meta-analysis in the context of a rapidly progressing pandemic should be intensified, determining the methodological quality of the study design and the level of bias in the analysis.^(12)^A critical view is important to know the advantages and disadvantages of using preprints, rapid reviews, living systematic reviews and scoping reviews. Considering the articles analyzed in the study by Pires et al.,^(1)^ it was found that, although the tool used identified some meta-analyses of better quality, most studies had mediocre quality and would bring more damage than benefits when used for decision-making. Literature reviews are crucial to identify the state-of-the-art and evidence gaps of the study object and enable targeting the specificities of study methodologies to offer more accurate answers, especially in the unprecedented condition of COVID-19.
